# Exploiting Mixed Waste Office Paper Containing Lignocellulosic Fibers for Alternatively Producing High-Value Succinic Acid by Metabolically Engineered *Escherichia coli* KJ122

**DOI:** 10.3390/ijms26030982

**Published:** 2025-01-24

**Authors:** Walainud Congthai, Chutchawan Phosriran, Socheata Chou, Kanyarat Onsanoi, Chotika Gosalawit, Kuan-Chen Cheng, Kaemwich Jantama

**Affiliations:** 1 Metabolic Engineering Research Unit, School of Biotechnology, Institute of Agricultural Technology, Suranaree University of Technology, 111 University Avenue, Suranaree Subdistrict, Muang District, Nakhon Ratchasima 30000, Thailand; noeywalainud1234@gmail.com (W.C.); phosriran.c1994@gmail.com (C.P.); socheatachou1@gmail.com (S.C.); kanyarat.ons88@gmail.com (K.O.); gosarawit2529@gmail.com (C.G.); 2 Institute of Food Science and Technology, College of Bioresources and Agriculture, National Taiwan University, Taipei 10617, Taiwan; kccheng@ntu.edu.tw; 3 Department of Medical Research, China Medical University Hospital, China Medical University, 91, Hsueh-Shih Road, Taichung 40447, Taiwan

**Keywords:** *Escherichia coli*, metabolic engineering, mixed waste office paper, succinic acid, fermentation, optimization, lignocelluloses

## Abstract

Succinic acid is applied in many chemical industries in which it can be produced through microbial fermentation using lignocellulosic biomasses. Mixed-waste office paper (MWOP) containing lignocellulosic fibers is enormously generated globally. MWOP is recycled into toilet paper and cardboard, but the recovery process is costly. The reuse of MWOP to alternatively produce succinic acid is highly attractive. In this study, pretreatment of MWOPs with 1% (*v*/*v*) H_2_SO_4_ at 121 °C for 20 min was found to be optimal. The optimal conditions for the enzymatic hydrolysis of H_2_SO_4_-pretreated MWOP (AP-MWOP) were at 50 °C, with cellulase loading at 80 PCU/g AP-MWOP. This resulted in the highest glucose (22.46 ± 0.15 g/L) and xylose (5.11 ± 0.32 g/L). Succinic acid production via separate hydrolysis and fermentation (SHF) by *Escherichia coli* KJ122 reached 28.19 ± 0.98 g/L (productivity of 1.17 ± 0.04 g/L/h). For simultaneous saccharification and fermentation (SSF), succinic acid was produced at 24.58 ± 2.32 g/L (productivity of 0.82 ± 0.07 g/L/h). Finally, succinic acid at 51.38 ± 4.05 g/L with yield and productivity of 0.75 ± 0.05 g/g and 1.07 ± 0.08 g/L/h was achieved via fed-batch pre-saccharified SSF. This study not only offers means to reuse MWOP for producing succinic acid but also provides insights for exploiting other wastes to high-value succinic acid, supporting environmental sustainability and zero-waste society.

## 1. Introduction

Succinic acid is considered one of the most important building block chemicals and has been identified as one of twelve potential future chemical structures by the U.S. Department of Energy to be produced from biomasses. It is a valuable chemical with a wide range of industrial applications and is also seen as a potential precursor for the production of other important key chemicals, including food additives, biodegradable polymers, fragrances, flavoring agents, fungicides and herbicides [1]. For this reason, its commercial demand is expected to increase to 768 million metric tons, with a CAR of 27.4% by the year 2025 [2]. Currently, succinic acid is produced primarily through chemical processes involving the use of n-butane as the starting material to create maleic anhydride, which is then hydrogenated and hydrated to yield succinic anhydride and finally succinic acid. These processes require high-energy and expensive catalysts, including Ni/Zr/Al/Si alloys, to achieve highly selective conversion of succinic anhydride to succinic acid [3]. The reactions also generate greenhouse gases, which are known to be environmentally unfriendly. In contrast, the biological process of microbial fermentation is presently an attractive method for producing succinic acid to avoid expensive manufacturing costs and environmental problems from the production of succinic acid by petrochemical processes.

The paper industry is rapidly expanding due to the enormous demand for papers used in many everyday documents, contributing to an annual waste of approximately 100 million tons [4]. Wastepaper consists of 50–80% cellulose, 5–15% hemicellulose and a negligible amount of lignin [5]. Approximately 400 million tons of wastepaper are produced annually; however, only 65% of the wastepaper is recovered because of the low quality of the paper [6]. The remaining waste is inefficiently burned or sent to landfills, causing environmental issues. Alternatively, most waste office paper is recycled into toilet paper and cardboard, but the recovery process by this means is costly due to the requirements of a more complex production process and more additional steps than those of brand-new papers [7]. To reduce expenses, waste paper has been broken into fermentable sugars as substrates for the production of cellulase enzymes and other biofuels and bioproducts instead [8]. Byadgi and Kalburgi [9] also successfully utilized waste newspaper to produce bioethanol with a yield of 6.91% using *Saccharomyces cerevisiae*. Additionally, D-lactic acid was produced from sugars derived from waste office paper and newspaper hydrolysates by *Escherichia coli* JH13, achieving concentrations of 35.86 ± 0.62 g/L and 25.64 ± 0.48 g/L, respectively [10].

**Figure 1:**
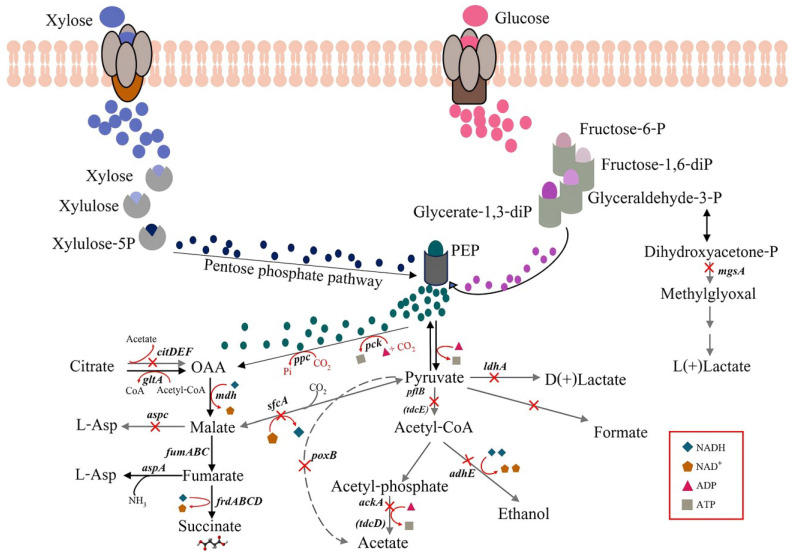
A schematic illustration of succinic acid production pathway by *E. coli* KJ122. The cross signs represent the deletion of certain genes (adapted from [11]).

The challenge for producing succinic acid by microorganisms is the high production costs due to expensive carbon substrates such as glucose, which can counteract the market price of succinic acid. However, succinic acid production through microbial fermentation can be cost-effective once renewable feedstocks, including lignocellulosic biomasses, are utilized by microbes as starting materials during the process. Research on improving succinic acid production by renewable feedstocks has focused on *E. coli* strains. *E. coli* KJ122 was developed through a combined strategy of genetic engineering and evolutionary engineering. *E. coli* KJ122 was metabolically engineered for improving the succinic acid production by eliminating genes, including *ldhA, adhE*, *ackA*, *focA-pflB*, *mgsA*, *poxB*, *tdcDE*, *citF*, *aspC* and *sfcA,* to eliminate or decrease by-products (Figure 1). It has shown the ability to produce high levels of succinic acid, reaching 80 g/L with a molar yield of 1.46 mol/mol glucose consumed and an average volumetric productivity of 0.9 g/L/h [11]. Additionally, *E. coli* KJ122 has been shown to produce impressive amounts of succinic acid from various lignocellulosic biomasses, including sugarcane bagasse, rice straw, oil palm empty fruit bunch, and cassava pulp [12]. However, no study has employed waste office paper for succinic acid production. Hence, the feasibility of producing bio-succinic acid through microbial fermentation via waste office paper through appropriate optimization techniques needs to be explored. Therefore, this study developed an efficient process for succinic acid production by the *E. coli* KJ122 strain from abundant waste office paper, a significant global waste material that is often discarded or burned without further use. First, the efficient pretreatment process of mixed waste office paper (MWOP) was investigated to efficiently remove impurities, including inks, which can inhibit microbial growth during succinic acid production. Consequently, the fermentation process for producing succinic acid by *E. coli* using pretreated MWOP was then optimized to achieve high succinate concentrations, yields, and productivity. This may facilitate further developments to produce succinic acid from MWOP on large industrial scales. In this context, valorizing MWOP to produce succinic acid may be an attractive alternative option for waste management and reducing environmental pollution.

## 2. Results and Discussion

### 2.1. Pretreatment of MWOP with Diluted H_2_SO_4_

Hayat et al. [13] reported that waste papers usually contain 40% (*w*/*w*) cellulose, 32.5% (*w*/*w*) hemicellulose, and 22.5% (*w*/*w*) lignin. However, MWOPs used in this study contained 81.43 ± 1.90% (*w*/*w*) cellulose, 2.43 ± 2.27% (*w*/*w*) hemicellulose, 0.69 ± 0.04% (*w*/*w*) lignin, and 7.95 ± 0.18% (*w*/*w*) ash based on proximal analysis. This suggests that MWOPs contain cellulose and hemicellulose fibers that can be broken down by combined chemical and enzymatic hydrolysis to obtain released fermentable sugars, mainly glucose and xylose, to be fermented to produce valuable biochemicals. Therefore, MWOP may serve as an alternative substance with renewable potential as a feedstock for succinic acid production. Mansy et al. [14] demonstrated the effectiveness of various chemical pretreatments for waste papers using acids, bases, and hot water. However, they revealed that pretreatment with bases or alkaline agents, in addition to acetic acid and hot water pretreatments, was a noneffective pretreatment for waste office paper because there was no significant increase in the number of released glucose units even after additional physical pretreatments involving autoclaving, microwaving, and sonication [15]. In contrast, strong acid pretreatment, especially with H_2_SO_4_, is one of the most widely used options because it does not require a recovery step, making it favorable for industrial applications because of its higher monosaccharide conversion yield than those of other strong acids, including HCl and H_3_PO_4_ [15]. The enzymatic hydrolysis of cellulose and hemicellulose fibers in H_2_SO_4_-pretreated MWOPs can be more effective not only in reducing the recalcitrance of those fibers but also in preventing the formation of microbial inhibitors, including furfural and 5-hydroxymethyl furfural (5-HMF), derived from sugar degradation.

**Figure 2:**
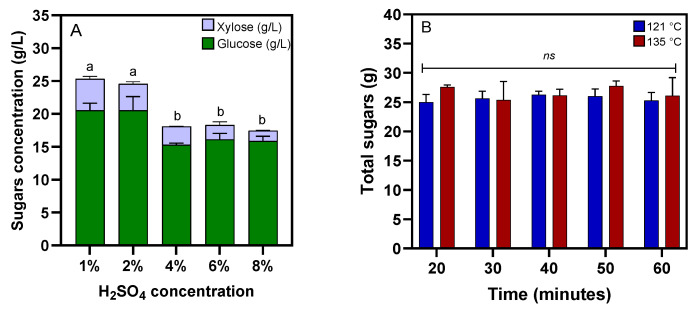
Effects of different H_2_SO_4_ concentrations for pretreatment of MWOP at 121 °C for 30 min (**A**) and effect of pretreatment time for pretreatment of MWOP at 121 °C and 135 °C (**B**) on total released fermentable sugars (glucose and xylose) from the acid-pretreated MWOP after enzymatic hydrolysis by crude cellulase. Each column represents the mean ± SD from three independent replicates. The different alphabet (a or b) above each column indicates statistical significance of differences between the mean values of the total released sugars among different treatments (*p* < 0.05). Data considered not significant among different treatments are marked *ns* (*p* > 0.05).

In this study, H_2_SO_4_ solutions at concentrations of 1%, 2%, 4%, 6%, and 8% (*v*/*v*) were used for the pretreatment of MWOP at 121 °C for 30 min. Subsequently, the total amount of sugars released from AP-MWOP was investigated via enzymatic hydrolysis by crude cellulase at 80 PCU/g AP-MWOP. The results indicated that the enzymatic hydrolysis of 50 g/L AP-MWOP pretreated with diluted H_2_SO_4_ at 1%, 2%, 4%, 6%, and 8% (*v*/*v*) resulted in total liberated sugars of 25.32 ± 1.42, 24.56 ± 2.37, 18.07 ± 0.27, 18.28 ± 0.41, and 17.44 ± 0.77 g/L, respectively, with a conversion yield of up to 50.64 ± 0.05% (Figure 2A). The pretreatment of MWOP with H_2_SO_4_ at concentrations greater than 2% (*v*/*v*) adversely affected the total number of sugars released. The high percentage of H_2_SO_4_ solution used for pretreatment likely caused a decrease in the amount of sugar released due to a loss of hemicellulose structures into the black liquor fraction under extremely high acidity during the pretreatment step. This is similar to the findings of Kumar et al. [16], in which the effectiveness of the diluted H_2_SO_4_ pretreatment of most lignocellulosic materials was only achieved when its concentration was less than 4% (*v*/*v*). Rocha et al. [17] and Nair et al. [18] reported that pretreatment of waste office paper with 1% (*v*/*v*) H_2_SO_4_ increased the cellulose content with no detectable levels of furfural or 5-HMF, thus indicating that no detoxification is needed before its subsequent use for bioethanol production by *Spathapora passalidarum* and biodiesel production by *Cryptococcus curvatus*. Da Mota and Gouveia [19] also pretreated waste office paper with 1% (*v*/*v*) H_2_SO_4_ at 50 °C for 3 h to achieve a maximum glucose concentration of 23 g/L. In contrast to H_2_SO_4_, HCl solution at high concentrations was used to pretreat waste office paper. For example, HCl at a concentration of 15% (*v*/*v*) was utilized for the pretreatment of 60 g/L waste office paper. However, the maximum amount of liberated glucose was only 15.7 g/L, with a conversion yield of 26.2% [14]. It is likely that the use of high HCl concentrations for pretreating waste office paper may not be suitable for large-scale applications since highly concentrated acids are hazardous, toxic, and corrosive, thus requiring corrosion-resistant reactors that also increase the capital investment or even operating cost of equipment used for high acid-catalyzed pretreatment. Additionally, NaOH pretreatment was used to demonstrate the pretreatment of waste office paper. On the other hand, Mokatse and van Wyk [20] and Ojewumi et al. [21] reported the utilization of 1.0% (*w*/*v*) NaOH to pretreat waste office paper. However, both studies showed that liberated sugar concentrations of 5.7 g/L and 0.37 g/L were achieved only from NaOH-pretreated waste office paper. Both studies showed that alkaline pretreatment may not be suitable for pretreating MWOP. These data suggested that the 1% (*v*/*v*) H_2_SO_4_ solution was more suitable for pretreating MWOP than the other pretreatment methods were.

H_2_SO_4_ is typically used for pretreatment at concentrations between 0.2% and 2.5% (*v*/*v*) and at high temperatures ranging from 130 °C to 210 °C to increase the digestibility of pretreated substrates but to reduce the pretreatment time [22]. In this experiment, the utilization of higher temperatures at 135 °C for 30 min was therefore investigated with the use of 1% (*v*/*v*) H_2_SO_4_ pretreatment. The results revealed that the total amount of sugar released at the insignificant level of 26.38 ± 4.06 g/L, which was only 4.19% greater than that obtained at 121 °C, was observed after enzymatic hydrolysis. An increase in the pretreatment time during autoclaving at 135 °C greater than 30 min did not even improve the enzymatic digestibility of the H_2_SO_4_-pretreated MWOP (AP-MWOP) by crude cellulase. The total liberated sugar levels were also comparable even when the pretreatment time was prolonged to 60 min (Figure 2B). Pretreatment at 135 °C for only 20 min inversely enhanced the effectiveness of enzymatic hydrolysis by increasing the released sugar level to 27.58 ± 0.34 g/L, which was approximately 8.92% greater than that obtained at 121 °C for 20 min. However, the levels of the sugars liberated from both conditions were not significantly different. Therefore, pretreatment of the diluted H_2_SO_4_ mixture of MWOP with 1% (*v*/*v*) H_2_SO_4_ at 121 °C for 20 min was determined to be the most suitable condition. With this optimal condition, the recovery yield of AP-MWOP was only 0.65 ± 0.04 g/g MWOP provided. Additionally, AP-MWOP contained 83.20 ± 0.29% (*w*/*w*) cellulose, 4.48 ± 1.43% (*w*/*w*) hemicellulose, 0.73 ± 0.03% (*w*/*w*) lignin, and 7.32 ± 0.47% (*w*/*w*) ash. This suggested that AP-MWOP possessed most of the cellulose fibers with low levels of hemicellulose and lignin. Díaz-Blanco et al. [23] studied the diluted H_2_SO_4_ pretreatment of *Agave lechuguilla* at temperatures ranging from 160 °C to 200 °C with acid concentrations between 0.5% and 1.5% (*v*/*v*). The optimal conditions for pretreating agave stems were 180 °C and 1.24% (*v*/*v*) H_2_SO_4_ with 10% (*w*/*v*) biomass loading. Under these conditions, the recovery rates of hemicellulose sugars and glucose reached maximum at 87% and 68%, respectively. Our results and other findings suggested that the acid concentration and pretreatment temperature had a significant effect on cellulose and hemicellulose recovery and the formation of degradation products during pretreatment, whereas the pretreatment time had a lesser effect. However, these findings contradict those of Martín et al. [24], who reported that optimal pretreatment conditions for high enzymatic conversion of cellulose in starch-depleted cassava stems were achieved at 195 °C with a relatively low concentration of 0.6% (*v*/*v*) H_2_SO_4_ and a pretreatment time of more than 35 min. The maximum cellulose breakdown at 72% was obtained at the pretreatment time of 50 min. However, these authors suggested that H_2_SO_4_ pretreatment at high temperatures and longer times may have disadvantages in which high energy input is required while high hemicellulose degradation is also acquired.

**Figure 3:**
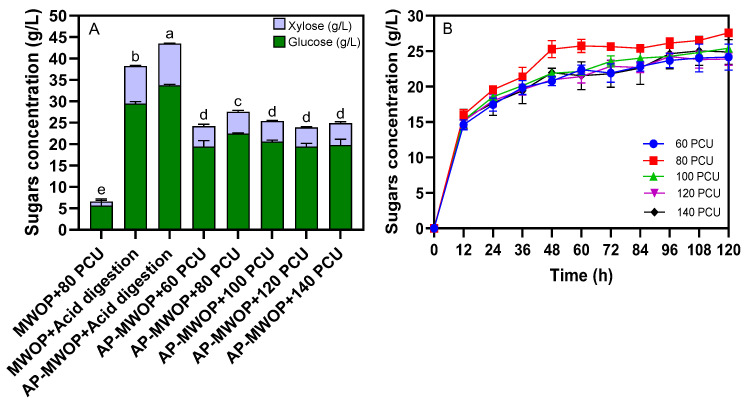
Effect of strong acid digestion and crude cellulase loadings (PCU: protein centered unit) used during enzymatic hydrolysis of 50 g/L MWOP (untreated) and AP-MWOP on total released sugars (glucose and xylose) (**A**), and total released sugars from 50 g/L AP-MWOP hydrolyzed with different crude cellulase loadings at different time intervals (**B**). Each column represents the mean ± SD from three independent replicates. The different alphabet above each column indicates statistical significance of differences between the mean values of the total released sugars among different treatments (*p* < 0.05).

Figure 3A shows the effects of pretreatment on the total released sugars from 50 g/L untreated MWOP and AP-MWOP by strong acid digestion and cellulase hydrolysis. The results revealed significant differences in the levels of sugars released from untreated MWOP (38.21 ± 0.49 g/L with a yield of 0.76 ± 0.01 g sugars/g MWOP) and AP-MWOP (43.47 ± 0.20 g/L with a yield of 0.86 ± 0.00 g/g AP-MWOP) after strong acid digestion. For cellulase hydrolysis, AP-MWOP (27.58 ± 0.34 g/L with a yield of 0.55 ± 0.00 g/g AP-MWOP) offered a substantially higher level of released sugars than did untreated MWOP (6.74 ± 0.61 g/L with a yield of 0.13 ± 0.03 g/g MWOP). This indicated that pretreatment with dilute H_2_SO_4_ had a greater beneficial effect on the enzymatic hydrolysis of AP-MWOP. On the basis of the results of this study, pretreatment of the diluted H_2_SO_4_ mixture of MWOP with 1% (*v*/*v*) H_2_SO_4_ at 121 °C for 20 min was determined to be the most suitable condition for preparing AP-MWOP for subsequent enzymatic hydrolysis to obtain the highest fermentable sugar level for succinic acid production. This was supported by Guo et al. [25], who reported that acid pretreatment involves the breakdown or attack of intramolecular and intermolecular bonds between cellulose, hemicellulose, and lignin by hydronium ions, affecting the porosity of the substrate and the accessibility of cellulase.

### 2.2. Optimal Cellulase Loading for the Enzymatic Hydrolysis of AP-MWOP

In this study, the effect of enzyme loading on total liberated sugars was assessed to minimize the level of crude cellulase used during the enzymatic hydrolysis of AP-MWOP. Based on proximal analysis, AP-MWOP contained 83.20 ± 0.29% (*w*/*w*) cellulose, 4.48 ± 1.43% (*w*/*w*) hemicellulose, 0.73 ± 0.03% (*w*/*w*) lignin, and 7.32 ± 0.47% (*w*/*w*) ash. Since AP-MWOP contained primarily cellulose with a small amount of hemicellulose, the commercialized VRE P3 crude cellulase possessing combined activities of mostly cellobiohydrolase (exoglucanase) and endoglucanase was chosen to hydrolyze AP-MWOP. According to the manufacturer’s instructions, both of which are capable of hydrolyzing 1,4-β-D-glycosidic bonds found in cellulose and hemicellulose fibers residing in lignocellulosic materials, including AP-MWOP. Additionally, the crude cellulase contains a trace amount of xylanase that facilitates digesting a small amount of xylan in the hemicellulose compartment. The crude cellulase was used to hydrolyze 50 g/L AP-MWOP at enzyme loadings of 60, 80, 100, 120, and 140 PCU/g AP-MWOP. The enzyme loading analysis revealed total released sugar concentrations of 24.17 ± 1.82 g/L, 27.58 ± 0.34 g/L, 25.40 ± 0.45 g/L, 23.88 ± 0.88 g/L, and 24.89 ± 1.74 g/L, respectively (Figure 3A). The results revealed that increasing the enzyme concentration to 80 PCU/g AP-MWOP resulted in significantly greater production of fermentable sugars (glucose and xylose), with the highest sugar yield of 55.16%. However, a further increase in cellulase loading did not increase the total sugar yield. This suggested that the use of higher cellulase loadings rapidly accumulated high amounts of cellobiose and glucose during enzymatic hydrolysis. This caused the inhibition of cellulase activities of both endoglucanase and β-glucosidase, Hsieh et al. [26], thus resulting in the alleviation of total liberated monosaccharides but increasing cellobiose levels instead. Therefore, the lowest level of enzyme loading that sufficiently and efficiently hydrolyzed AP-MWOP to the highest fermentable sugars was preferable. Furthermore, Figure 3B shows the hydrolysis rate of AP-MWOP at different cellulase loadings over 120 h. Enzymatic hydrolysis rapidly increased within 12 h of incubation, as reflected by a sharp increase in the total released sugar levels in all cellulase loadings. However, the hydrolysis rates suddenly decreased after 12 to 48 h. No significant increases in total released monosaccharide sugars were noted after 48 h. The utilization of 80 PCU/g AP-MWOP with a sugar yield of 55.16% was thus found to be optimal, as reflected by the highest level of total released sugars and the highest hydrolytic rate compared with those of other cellulase loadings.

Annamalai et al. [27] demonstrated that the sugar yield substantially increased with 1–3% (*w*/*w*) total solid enzyme loading but started to decrease with 4% (*w*/*w*) solid enzyme loading for office paper. The maximum glucose yield (23.48 g/L) occurred at 3% (*w*/*w*) enzyme loading, and the minimum glucose yield (8.82 g/L) occurred at 1% (*w*/*w*) enzyme loading. These findings may suggest that high enzyme loading diminishes the sugar yield as the consistency of the substrates suddenly increases at high enzyme loadings, which influences the mixing homogeneity and mass transfer of the enzymes and causes feedback inhibition via the increased concentration of sugars [17]. AI-Battashi and Sivakumar [28] demonstrated PHB production by a *Cupriaviduc necator* via the SSF process via the enzymatic hydrolysis of 100 g/L H_2_O_2_-pretreated waste office paper (OPHT) with 55.5 FPU cellulase (Sigma, Darmstadt, Germany, C2730)/g OPHT and 37.5 CBU β-glucosidase (Sigma, 49,291)/g OPHT. However, the maximum PHB concentration of 4.29 g/L was only achieved with a yield of 0.043 g PHB/g OPHT. This finding implied that the high cellulase loading provided in the study did not guarantee the effectiveness of the enzymatic hydrolysis to achieve high levels of fermentable sugars from OPHT to produce PHB at high concentrations and yields. Brummer et al. [29] performed enzymatic hydrolysis of waste office paper to achieve a high sugar yield of 18.8% with 2% (*w*/*w*) cellulase complex (NS50013, Novozymes A/S, Bagsvaerd, Denmark) and 0.2% (*w*/*w*) β-glucosidase (NS50010, Novozymes A/S, Bagsvaerd, Denmark). Additionally, they recommended the utilization of high enzyme loadings of up to 10% (*w*/*w*) cellulase complex and 1% (*w*/*w*) β-glucosidase for hydrolysis to achieve a twofold higher sugar yield, thus ensuring industrial application for bioethanol production. Park et al. [30] also demonstrated the use of the commercial Acremonium cellulase AUS0301 for hydrolyzing unpretreated waste office paper. They demonstrated that cellulase at 20.6 FPU/g office paper was required to achieve a hydrolytic yield of up to 50%. However, they also proposed that a higher cellulase loading of 105 FPU/g office paper could be used to obtain a sugar conversion yield of up to 80% to effectively produce bioethanol on a large scale. Hossain et al. [31] further revealed that the maximum glucose content obtained was 9.75 g/L from acid-treated waste paper (50% glucose yield) after 5 days of enzymatic hydrolysis, with up to 250 FPU/g of acid-pretreated waste paper. Mansy et al. [14] reported that the glucose level increased from 2.98 g/L to 5.01 g/L during the digestion of HCl-pretreated waste office paper when the cellulase loading was increased from 1% to 7% (*w*/*w*). When 9% (*w*/*w*) cellulase was used, the glucose concentration decreased to 4.07 g/L after 24 h of incubation. Additionally, they reported that the incubation time was 72 h, resulting in the highest release of glucose units at 15.72 g/L when 5% (*w*/*w*) cellulase enzyme was used, in which the cellulase loading greater than 5% (*w*/*w*) dramatically decreased. Furthermore, da Mota and Gouveia [19] reported that using the lowest cellulase loading (Novozymes 188) of 5.2 FPU/g and 199 CBU/g β-glucosidase with a high solid mass of waste office paper at 75 g/L was optimal for achieving the highest glucose level among the enzyme loadings tested. These findings confirm that excessive amounts of cellulase enzymes decrease the amount of glucose released. This may imply that an enzyme loading higher than a certain critical value does not improve hydrolysis, since the excess enzyme adsorbed into the substrate restricts the diffusion process through the structure. With the abovementioned information, several previous works showed that high liberated levels of fermentable sugars from pretreated waste office papers were achieved by the use of commercially high-cost cellulase with high enzyme loadings to obtain high levels of fermentable sugars for the subsequent production of bioproducts, but this strategy may increase the cost of the production of bioproducts via fermentation. Compared with our results, the use of crude cellulase at a lower enzyme loading for hydrolyzing MWOP to obtain a relatively high sugar yield compared with those obtained from other previous studies may therefore reduce the cost of succinic acid production at the industrial scale.

Enzymatic hydrolysis by cellulase has advantages over chemical or thermochemical methods to deconstruct the recalcitrance of MWOP due to its simple equipment requirements. Enzymatic hydrolysis is performed at lower temperatures (45–50 °C) and moderate acidic pH at 5.0 compared to those of chemical or thermochemical digestion. This reduces energy consumption and the risk of equipment corrosion. The enzymatic hydrolysis process can operate in conjunction with fermentation platforms [1]. In contrast, chemical or thermochemical hydrolysis involves high temperatures and pressure, high energy input, and low pH levels, which can lead to equipment corrosion and the need for costly recovery processes. Additionally, toxic inhibitors derived from the degradation of sugars like 5′-HMF, furfural, and acetic acid are often generated during chemical or thermochemical processes. These compounds significantly cause adverse effects by inhibiting enzymatic hydrolysis and microbial growth during fermentation processes [18,19]. Enzymatic hydrolysis was then employed for saccharifying fermentable sugars from AP-MWOP to subsequently produce succinic acid by fermentation in further experiments.

### 2.3. Succinic Acid Production from AP-MWOP via SHF by E. coli KJ122

**Figure 4:**
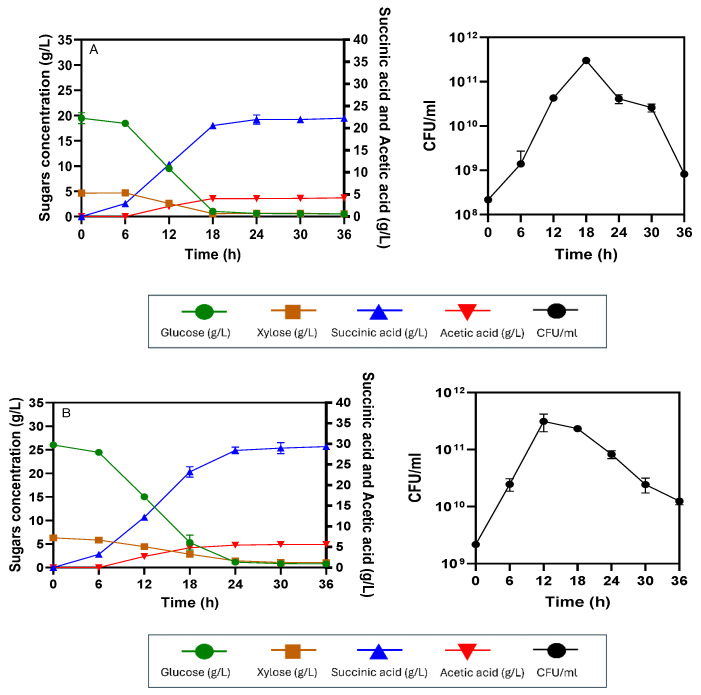

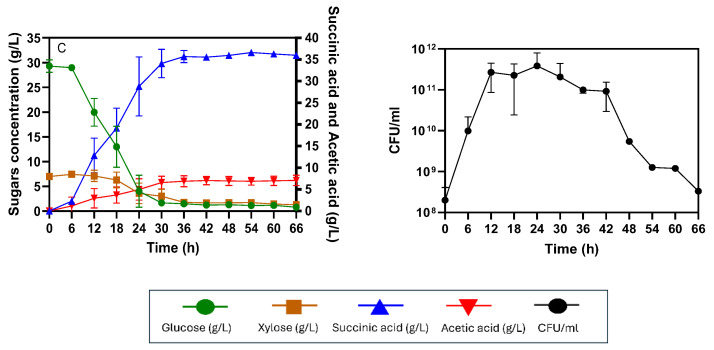
Succinic acid production via SHF by *E. coli* KJ122 using different concentrations of AP-MWOP. (**A**) 50 g/L AP-MWOP, (**B**) 70 g/L AP-MWOP, and (**C**) 100 g/L AP-MWOP. Three independent replications were performed on each test, and the means were reported with the standard deviation (SD).

The production of succinic acid from AP-MWOP at different initial concentrations via the SHF process with a 2.5 L working volume in a 5 L fermenter using *E. coli* KJ122 was investigated to evaluate the effects of the AP-MWOP concentration on succinic acid production. For 50 g/L AP-MWOP, initial glucose at a concentration of 19.47 ± 1.06 g/L was slowly consumed in the first 6 h of incubation, whereas no initial xylose at 4.64 ± 0.17 g/L was utilized. After 6 h of incubation, glucose was rapidly consumed by *E. coli* KJ122, while xylose was also co-utilized. Both glucose and xylose were exhausted within 18 h, with consumption rates of 0.79 ± 0.04 and 0.24 ± 0.03 g/L/h, respectively. Succinic acid production increased rapidly between the 6–18 h incubation period, corresponding to rapid glucose consumption, indicating that succinic acid is a growth-associated product. The cell viability approached the exponential phase within 18 h and then entered the death phase because no sugars remained in the fermentation broth to support cell growth and maintenance. After 18 h, succinic acid production slightly increased and did not improve after 24 h because of substrate exhaustion (Figure 4A). Succinic acid was produced at a level of 21.79 ± 0.65 g/L, with a yield of 0.90 ± 0.02 g/g sugar utilized, 0.43 ± 0.01 g/g AP-MWOP provided, and a productivity of 0.90 ± 0.02 g/L/h. Acetic acid was produced at a level of 4.10 ± 0.10 g/L. For 70 g/L AP-MWOP (initial contents of 26.03 ± 0.06 g/L glucose and 6.30 ± 0.06 g/L xylose), the production of succinic acid and acetic acid was 28.19 ± 0.98 g/L and 5.36 ± 0.31 g/L, respectively. Glucose was fully consumed at a rate of 0.86 g/L/h within 30 h. Xylose consumption started at 18 h and was complete within 30 h at a consumption rate of 0.21 g/L/h. Succinic acid was substantially produced between 6 h and 24 h, and no increase in succinic acid production was observed after 24 h, even when the incubation time was prolonged to 36 h (Figure 4B). Succinic acid production reached its maximum at 24 h, with yields of 0.87 ± 0.03 g/g sugar utilized, 0.40 ± 0.02 g/g AP-MWOP provided, and a productivity of 1.17 ± 0.04 g/L/h. There was an 8.4% increase in productivity, whereas the yields were not significantly different from those of 50 g/L AP-MWOP (Table 1). For 100 g/L AP-MWOP (initial contents of 29.29 ± 1.25 g/L glucose and 6.97 ± 0.34 g/L xylose), glucose consumption started at 6 h and was prolonged until 36 h of incubation. After that, glucose still remained at a concentration of approximately 1 g/L until 66 h without further utilization. Xylose consumption began at 12 h and even slowly continued until 36 h. Xylose at a concentration of 2 g/L remained after 36 h until the end of fermentation. Succinic acid production was quite stable after 36 h but reached the highest level at 54 h, reaching 36.62 ± 0.02 g/L, along with the production of acetic acid at 7.15 ± 1.12 g/L (Figure 4C).

However, the cells entered the death phase beginning at 24 h, corresponding to the depletion of sugars. Although the succinic acid concentration was greater than those of 50 and 70 g/L AP-MWOP, an overall succinic acid yield and productivity of 0.36 ± 0.01 g/g AP-MWOP and 0.67 ± 0.01 g/L/h, respectively, were achieved (Table 1). The overall succinic acid yield decreased to 10%, while the productivity was also approximately 42.7% lower than that of 70 g/L AP-MWOP. However, the succinate conversion yield of 0.73 ± 0.01 g/g sugar utilized was still comparable. Notably, there was a decrease in the mixing efficiency due to high broth viscosity when the AP-MWOP concentration was increased. The nonhomogeneous mixing resulting from the high solid content, especially at 100 g/L AP-MWOP, caused inefficient and incomplete utilization of sugars, thus adversely affecting succinic acid production in terms of yield and productivity. This was likely due to an abrupt increase in mass and heat transfer during fermentation, reflected by the lack of further utilization of sugars after 30 h, resulting in the sugar remaining in the broth when 100 g/L AP-MWOP was used. Our findings were similar to those of Buyakoztekin and Buyukkileci [32]. They utilized organosolv-pretreated corncob at a high solid content of 105 g/L for succinic acid production through the SHF process by *A. succinogenes*. Succinic acid at a concentration of 12.7 g/L with a yield of 0.12 g/g pretreated corncob was achieved only because of inefficient and incomplete utilization of sugars with high broth viscosity. Therefore, the optimum concentration of AP-MWOP for succinic acid production via the SHF process is in the range of 50–70 g/L.

Increasing solid-substrate loading generally results in elevated amounts of waste office paper-derived glucose for the production of bioproducts. Nair et al. [8] demonstrated that increasing the level of pretreated waste office paper to 11 g/L significantly increased cellulase production by *Bacillus velzensis* ASN1. However, Mansy et al. [14] reported that providing the highest tested concentration of waste office paper did not result in the highest glucose concentration but even decreased bioethanol production at higher solid contents. Diaz-Blanco et al. [23] reported a poorer performance of bioethanol production with acid-pretreated agave stems at higher slurry viscosities. This resulted in a decrease in the glucose conversion yield and even bioethanol production of approximately 16%, even though the solid-substrate loading in the enzymatic hydrolysis of the whole slurry was slightly lower (44 g/L vs. 50 g/L). These results implied that a higher solid content of fermentation broth than the critical value unfavored the production of bioproducts by not only causing mixing inhomogeneity but also contributing to the presence of some inhibitory compounds in the slurry, which originated as a consequence of acid pretreatment, since toxic compounds, such as acetic acid, furfural, 5-HMF, formic acid and, especially, phenolic compounds from lignin, negatively affected enzymatic activity and microbial growth. This finding is supported by the findings of Park et al. [33], who reported that the production rate of L-(+)-lactic acid by *Rhizopus oryzae* may be inhibited by xylose derived from hemicellulose and that the yield may be inhibited by toxic compounds derived from waste office paper generated during the pretreatment process when higher concentrations of waste office paper are utilized.

### 2.4. Succinic Acid Production from AP-MWOP via Pre-Saccharification Batch SSF

**Figure 5:**
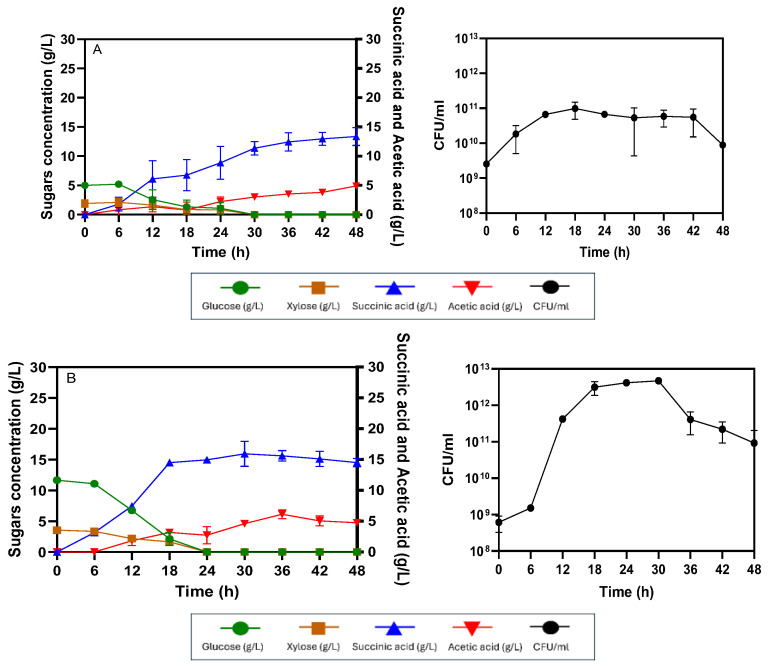

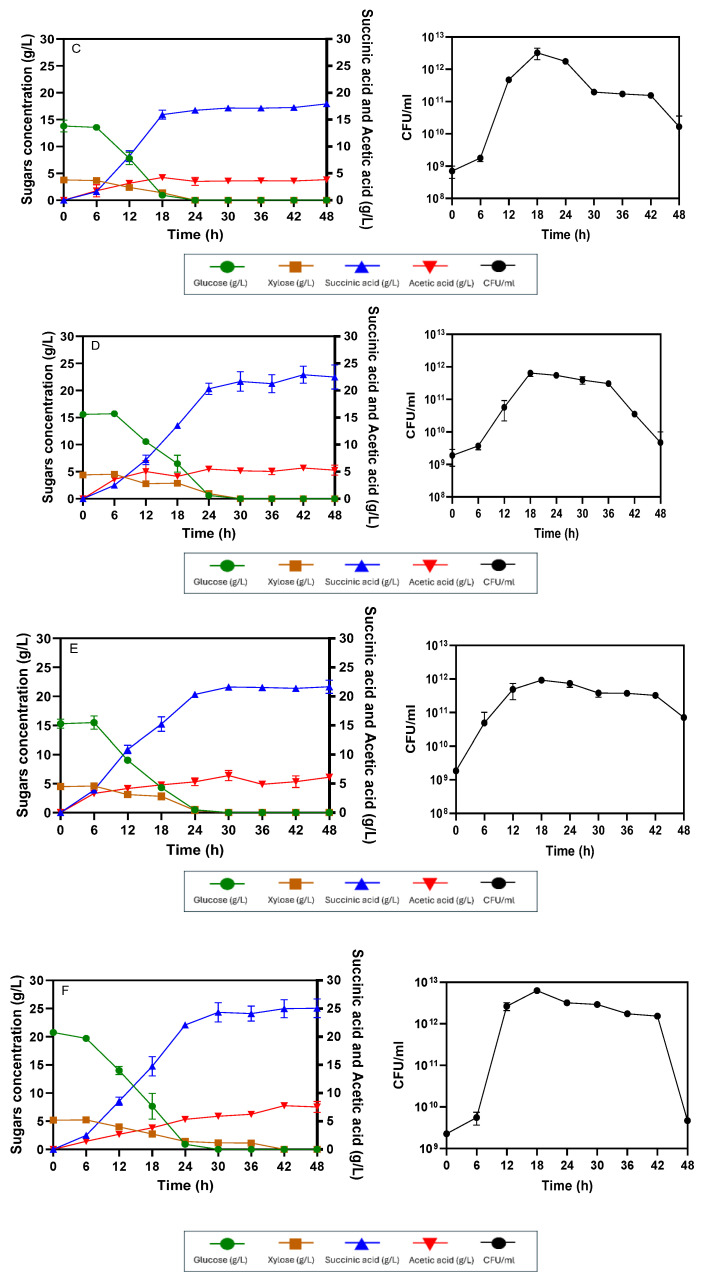
Succinic acid production via the SSF process with different pre-saccharification times. (**A**) 0 h, (**B**) 6 h, (**C**) 12 h, (**D**) 18 h, (**E**) 24 h with 50 g/L AP-MWOP, and (**F**) 18 h pre-saccharified batch SSF with 70 g/L AP-MWOP. Three independent replications were performed on each test, and the means were reported with the standard deviation (SD).

For the SSF process, sugar concentrations derived from substrates are usually kept low throughout the procedure. Sugars are slowly released from the pretreated substrate to avoid substrate and product inhibition during enzymatic hydrolysis and fermentation. Figure 5A shows the SSF process without pre-saccharification for producing succinic acid from AP-MWOP at 50 g/L AP-MWOP loading. Initially, initial sugar concentrations of 5.02 ± 0.03 g/L glucose and 2.10 ± 0.61 g/L xylose (7.12 ± 0.61 g/L total sugars) were found. Sugar consumption began after 6 h and gradually continued until 30 h, at which point all sugars were exhausted. The rates of glucose and xylose consumption were 0.33 ± 0.07 g/L/h and 0.11 ± 0.03 g/L/h, respectively. After 48 h, succinic acid production reached 14.51 ± 1.15 g/L, with a yield of 0.29 ± 0.02 g/g AP-MWOP provided and a productivity of 0.29 ± 0.02 g/L/h. The cells reached the exponential phase within 18 h, after which the cells entered the stationary phase. The culture eventually entered the death phase after 42 h of incubation. This was likely due to insufficient sugar availability, as cellulase activity was not sufficient to hydrolyze AP-MWOP to release fermentable sugars to maintain microbial growth. Typically, cellulase enzymes exhibit optimal activity at 50 °C and pH 5.0. However, the SSF process requires a lower temperature of 37 °C and a neutral pH of 7.0 to suit the optimal growth of *E. coli* KJ122. This discrepancy between the ideal conditions for cellulase activity (50 °C, pH 5.0) and for microbial fermentation (37 °C, pH 7.0) could result in a slower rate of hydrolysis during the SSF process [34]. Under these suboptimal conditions, succinic acid production via the SSF process was less efficient than that via the SHF process in terms of the reduction in the succinic acid concentration, yield, and productivity by 33.4%, 66.7%, and 32.6%, respectively (Table 1). Many studies have attempted to increase the mixing efficiency and enhance enzymatic hydrolysis during the SSF process. Wood et al. [35] reported that intermittent exposure of SSF processes to ultrasonic energy increased ethanol production from mixed waste office paper by 20% compared with nonultrasonic conditions. The ultrasonic energy unblemished the pulp in the waste office paper, thus reducing the viscosity of the slurry and facilitating increased accessibility of cellulase into the fibers, which could reduce the cellulase loadings used during the SSF process by 2-fold. However, they suggested that continuous exposure of the organism to ultrasound was bacteriostatic and decreased ethanol production in the long-term process. Additionally, Yang et al. [36] added the surfactant Gleditsia saponin to fermentation broth for lactic acid production by *Streptococcus thermophilus* via waste office paper with a high solid content via the SSF process to reduce cellulase loading while increasing mixing efficiency and preventing product inhibition. Although they suggested that saponin is a potential additive for developing an effective lactic acid production process, its price is quite high.

To address the issue of suboptimal conditions in SSF, the pre-saccharification SSF process was alternatively introduced to reduce the viscosity of the broth. For the 6 h pre-saccharified SSF, the initial glucose concentration was 7.71 ± 0.32 g/L, and the xylose concentration was 2.33 ± 0.22 g/L, yielding a total sugar concentration of 10.04 ± 0.55 g/L, representing a 29.08% increase in fermentable sugars compared with the non-saccharified SSF process. Glucose consumption occurred rapidly between 6 and 24 h at a rate of 0.49 ± 0.01 g/L/h, whereas xylose was slowly consumed at a rate of 0.15 ± 0.00 g/L/h over 24 h (Figure 5B). By the end of the process, the maximum succinic acid concentration was 15.36 ± 1.03 g/L, with a yield and productivity of 0.30 ± 0.02 g/g AP-MWOP provided and 0.42 ± 0.02 g/L/h, respectively. For the 12 h pre-saccharified SSF, the initial glucose concentration was 14.12 ± 1.34 g/L, and the xylose concentration was 3.85 ± 0.30 g/L, resulting in a total sugar concentration of 17.97 ± 1.64 g/L, representing a 60.37% increase compared with that of the non-saccharified SSF process. Glucose consumption was more rapid, particularly between 6 and 18 h, and was complete within 24 h of incubation, with a rate of 0.60 ± 0.04 g/L/h. Xylose consumption was slower at 0.16 ± 0.01 g/L/h but was also complete within 24 h (Figure 5C). This resulted in 17.15 ± 0.03 g/L succinic acid, with a yield and productivity of 0.34 ± 0.01 g/g AP-MWOP provided and 0.47 ± 0.02 g/L/h, respectively (Table 1).

For the 18 h pre-saccharified SSF, the initial glucose and xylose concentrations were 15.60 ± 0.07 g/L and 4.42 ± 0.21 g/L, respectively, which were 64.43% greater than those of the non-saccharified SSF process. Glucose consumption was even faster (0.65 ± 0.00 g/L/h) than that of the 12 h pre-saccharified SSF, occurring within 30 h, whereas xylose consumption was 0.18 ± 0.00 g/L/h (Figure 5D). Succinic acid was produced at 22.44 ± 1.93 g/L, with a yield of 0.44 ± 0.03 g/g AP-MWOP, which was comparable to that of the SHF process, and the productivity was 0.75 ± 0.04 g/L/h (Table 1). The succinic acid production in this case approached the comparable level observed in the SHF process, suggesting that the pre-saccharification time of 18 h was enough to allow cellulase to release fermentable sugars at a sufficient level to sustain microbial growth and succinic acid production. For the 24 h pre-saccharified SSF, the initial glucose concentration was 15.06 ± 0.96 g/L, and the xylose concentration was 4.48 ± 0.31 g/L, which was 63% greater than that of the non-saccharified SSF but not significantly different from that of the 18 h pre-saccharified SSF. Glucose consumption was similar to that in the 18 h pre-saccharification process, with a rate of 0.64 ± 0.03 g/L/h, thus completing within 30 h. Xylose consumption was 0.20 ± 0.01 g/L/h (Figure 5E). The yield and productivity of succinic acid were 21.39 ± 0.01 g/L, 0.42 ± 0.01 g/g AP-MWOP, and 0.71 ± 0.04 g/L/h, respectively (Table 1). Cell growth was consistent with sugar availability, showing a gradual decline after the exponential phase at 18 h and eventually entering the death phase due to the depletion of fermentable sugars. Succinic acid production under these conditions was also comparable to that obtained by the SHF process. It may be concluded that pre-saccharifying SSF could reduce the processing time and enhance the effectiveness of enzymatic hydrolysis. However, the rate of succinic acid production may vary depending on the duration of pre-saccharification, and enzyme activity remains a critical factor in determining overall succinic acid productivity.

The 18 h pre-saccharified SSF to produce succinic acid was then applied to 70 g/L AP-MWOP. The results revealed that sugar consumption was similar to that observed for 50 g/L AP-MWOP. Glucose consumption was rapid between 6 and 24 h, with a glucose consumption rate of 0.78 ± 0.01 g/L/h; thus, it was also complete within 30 h. Xylose consumption was slower, completing at 42 h, with a consumption rate of 0.12 ± 0.00 g/L/h (Figure 5F). The maximum level of succinic acid produced at 30 h was 24.58 ± 2.32 g/L, with a yield of 0.35 ± 0.03 g/g AP-MWOP provided and a productivity of 0.82 ± 0.07 g/L/h. Although succinic acid production was 12.80% lower than that of the SHF process using 70 g/L AP-MWOP, it represented an 8.7% increase compared with that of 50 g/L AP-MWOP. The results also suggested that the utilization of a higher concentration of 70 g/L AP-MWOP for succinic acid production via the batch SSF process may not yet improve the process performance in terms of concentration and productivity. A similar result was reported by Jampatesh et al. [37], who reported that the use of rice straw at concentrations greater than 70 g/L in SSF batches significantly decreased glucose utilization, succinate production and fermentation efficiency in *E. coli* AS1600a due to difficulties in mixing. Additionally, Akhtar et al. [38] revealed that succinic acid production was even lower in concentration (20.9 g/L) and yield (0.29 g/g) from 100 g/L NaOH-pretreated empty oil palm fruit bunches than in those obtained from this study when AP-MWOP at 50–70 g/L was used via the SSF process. Sangkharak [39] also revealed that ethanol production from pretreated office paper using *Saccharomyces cerevisiae* in the SSF process was only 2.1% (*v*/*v*), with a productivity of 0.58 g/L/h. This was due to a low level of liberated glucose at 2184.22 µg/L, even when a high cellulase loading of up to 28.14 FPU/g was provided.

In this study, no significant differences in initial sugar concentrations or succinic acid production were detected between the 18 h and 24 h pre-saccharified SSF processes. Therefore, the 18 h pre-saccharification time was considered the most suitable for the production of succinic acid by *E. coli* KJ122 via the SSF process, as it reduces fermentation time without sacrificing succinic acid production efficiency. At a 50 g/L substrate concentration, the total process time for the 18 h pre-saccharified SSF was 66 h compared with 96 h for the SHF. The energy consumption for the enzymatic hydrolysis was expected to decrease by 62.5% in the 18 h pre-saccharification period, probably reducing the production costs on an industrial scale. This suggests that the overall benefits of the 18 h pre-saccharified SSF, including a reduced fermentation time with energy savings and lower production costs, could make it more suitable for succinic acid production than the batch SHF process for industrial applications.

### 2.5. Effect of Agitation Speed on Succinic Acid Production via the SSF Process

**Figure 6:**
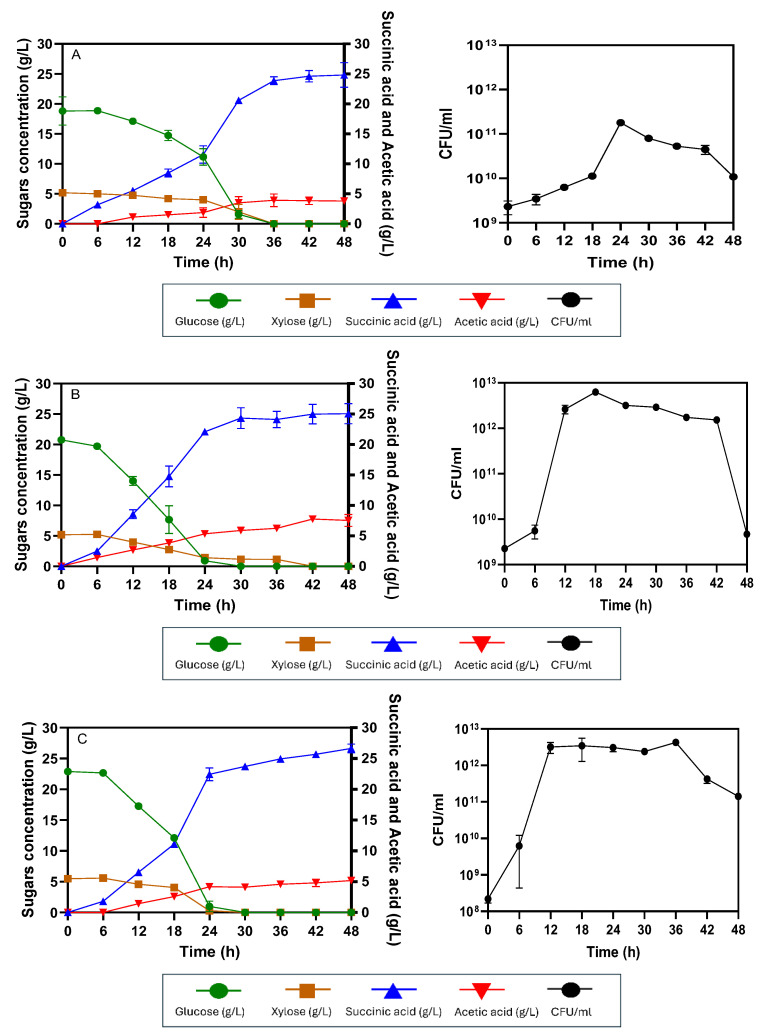
Effects of different agitation rates of 70 g/L AP-MWOP with 18 h pre-saccharification time on succinic acid production by *E. coli* KJ122. (**A**) 100 rpm, (**B**) 200 rpm, and (**C**) 300 rpm. Three independent replications were performed on each test, and the means were reported with the standard deviation (SD).

Mixing, an energy-intensive process, becomes particularly challenging in concentrated biomass slurries because of their high viscosity and non-Newtonian characteristics. Insufficient mixing can lead to mass and heat transfer limitations, as well as uneven enzyme distribution. Additionally, energy dissipation from stirring can influence particle size, further impacting enzymatic hydrolysis efficiency, which diminishes hydrolysis yields and decreases the performance of bioproduct production. In this study, the effects of various agitation speeds (100, 200, and 300 rpm) on succinic acid production were investigated (Figure 6).

At 100 rpm, glucose consumption was complete within 36 h at a rate of 0.72 ± 0.00 g/L/h, whereas xylose was slowly consumed at a rate of 0.15 ± 0.01 g/L/h, completing at 36 h (Figure 6A). This agitation speed provided 23.13 ± 0.52 g/L succinic acid with a yield of 0.74± 0.03 g/g sugar utilized and a productivity of 0.55 ± 0.01 g/L/h, along with 4.03 ± 0.77 g/L acetic acid. At 200 rpm, sugar consumption was faster, with glucose completely consumed within 30 h at a rate of 0.78 ± 0.10 g/L/h, whereas xylose was fully consumed at 42 h at a rate of 0.12 ± 0.00 g/L/h. The maximum succinic acid production occurred at 30 h, with a succinic acid production of 24.58 ± 2.32 g/L, a productivity of 0.82 ± 0.07 g/L/h and an acetic acid concentration of 7.25 ± 1.19 g/L (Figure 6B). At 300 rpm, the sugar consumption rates were similar to those observed at 200 rpm, with glucose completely consumed within 30 h at a rate of 0.89 ± 0.10 g/L/h and xylose fully consumed at the same rate of 0.21 ± 0.02 g/L/h. Succinic acid production was highest at 25.85 ± 1.03 g/L, with a yield of 0.80 ± 0.01 g/g and a productivity of 0.53 ± 0.01 g/L/h, along with 5.28 ± 0.40 g/L acetic acid (Figure 6C).

After the end of fermentation, succinic acid production was similar across all agitation speeds (100, 200, and 300 rpm). However, the glucose consumption rate was significantly lower at 100 rpm, likely because of insufficient mixing caused by the high viscosity of the fermentation broth, thus reducing the mass transfer efficiency [40]. Comparable succinic acid productivity was observed at 200 and 300 rpm. Therefore, succinic acid production at 200 rpm was considered optimal under these conditions. This result aligns with the shorter lag phase observed at 200 rpm, where succinic acid production was completed in approximately 30 h, compared with approximately 42 h at lower agitation speeds. Similar findings were reported by Oh et al. [41] suggested that insufficient CO_2_ dissolution in the bioreactor occurred at low agitation speeds, which had a negative effect on microbial cell growth rates. This finding also suggested that agitation speed impacts cell morphology, growth rates, metabolite production, and potential cell damage. Additionally, agitation is essential for ensuring proper mixing, facilitating mass transfer, and maintaining effective heat transfer within the system. Proper agitation promotes a uniform distribution of nutrients and gases, which is critical for optimizing microbial activity and overall fermentation performance. It facilitates mass transfer between different phases in culture and maintains uniform chemical and physical conditions [42].

### 2.6. Succinic Acid Production from AP-MWOP via Pre-Saccharified Fed-Batch SSF

**Figure 7:**
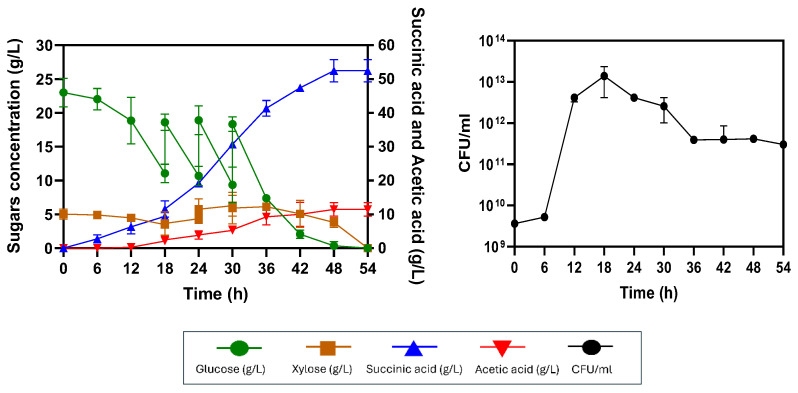
Succinic acid production by *E. coli* KJ122 via a pre-saccharified fed-batch SSF process with an initial concentration of 70 g/L AP-MWOP. Three independent replications were performed on each test, and the means were reported with the standard deviation (SD).

In this study, the strategy used to increase succinate production in *E. coli* KJ122 focused on fed-batch fermentation, a preferred industrial method in which essential nutrients for cell growth and product formation are added intermittently or continuously during batch operation. The fed-batch fermentation began with an 18 h pre-saccharified SSF using an initial substrate concentration of 70 g/L AP-MWOP. After pre-saccharification, the seed culture mixture was added to initiate succinic acid production. Substrates were fed whenever glucose levels dropped below 10 g/L. As shown in Figure 7, the initial glucose concentration was 23 ± 2.11 g/L, with 5.04 ± 0.76 g/L xylose. Since succinic acid production primarily utilizes glucose, substrate feeding was focused on maintaining the level of glucose to no less than 10 g/L. After 6 h, glucose was rapidly consumed until 18 h of incubation, and its remaining concentration was 11.05 ± 1.35 g/L. At this point, the sugar stock derived from AP-MWOP was added, thus increasing the glucose concentration to 20 g/L. Additional sugars were fed at 18, 24 and 36 h. At the end of the process, succinic acid at 51.38 ± 4.05 g/L was produced, with a comparable yield of 0.75 ± 0.05 g/g and a productivity of 1.07 ± 0.08 g/L/h. This represented a 52.16% increase in succinic acid production compared with that of the batch SSF operation. Cell availability showed a lag phase from 0 to 6 h, followed by an exponential phase from 6 to 18 h. After 18 h, cell growth was slow, possibly due to the increasing volume in the bioreactor after the addition of sugar, which reduced the cell density. Cell growth was stable between 36 and 42 h, after which the culture entered the death phase (Figure 7). Compared with other studies using fed-batch SSF, Zhong et al. [43] demonstrated succinic acid production by *Yarrowia lipolytica* from 65 g/L pretreated corncob. Succinic acid at a concentration of 45.4 g/L with a yield of 0.71 g/g was obtained. Grape stalks were also used to produce 40.2 g/L succinic acid, with a yield of 0.67 g/g produced by *A. succinogenes* [44]. Alexandri et al. [45] also utilized *A. succinogenes* to produce succinic acid from sugar beet pulp. However, only 30 g/L succinic acid was obtained, with yields and productivities of 0.8 g/g and 0.62 g/L/h, respectively. Additionally, carob pods are utilized by *A. succinogenes* 130Z to produce succinic acid [46]. However, the succinic acid concentration of 18.97 g/L was too low to apply in industrial applications. Therefore, the 18 h pre-saccharified fed-batch SSF process using AP-MWOP as a potential and sustainable substrate may be a suitable and cost-effective process for succinic acid production. This process should be further developed to produce succinic acid on an industrial scale.

## 3. Materials and Methods

### 3.1. Strains, Media, and Cultivations

*E. coli* KJ122 strain used in this study was stored at −80 °C at Suranaree University of Technology (SUT), Thailand. The seed culture was grown in Luria–Bertani (LB) medium containing 20 g/L glucose at 37 °C and 200 rpm in a 500 mL small vessel with a working volume of 350 mL for 16 h of incubation. The cultivation was maintained at pH 7.0 by automatically adding a mixture of 3 M K_2_CO_3_ and 6 M KOH at a 6:1 ratio. The seed culture mixture was then inoculated into fermentation medium at an initial OD_600_ of 0.1 (33.3 mg CDW/L). Anaerobiosis was quickly achieved during growth by the addition of bicarbonate to ensure a CO_2_ atmosphere. Low-salt AM1 medium supplemented with 100 mM KHCO_3_ and 1 mM betaine HCl was used as fermentation broth throughout this study for succinate production and strain maintenance [47].

### 3.2. Pretreatment of Mixed Waste Office Paper Hydrolysate (MWOP)

The MWOP was cut into pieces 3–5 cm in size and treated with diluted sulfuric acid at concentrations of 1%, 2%, 4%, 6%, and 8% (*v*/*v*) with a solid content of 10% (*w*/*v*). The mixture was then heated at 121 °C for 30 min in an autoclave. The optimal incubation time for autoclaving was also evaluated at different incubation periods of 20, 30, 40, 50, and 60 min.

The acid-pretreated MWOP (AP-MWOP) was washed with tap water until the drain tap water reached a neutral pH. The washed AP-MWOP was subsequently air-dried at 55 °C for 24 h until its moisture content reached approximately 5% (*w*/*w*). AP-MWOP pulping was used as a substrate for succinic acid production throughout this study. The total level of sugars released from AP-MWOP pretreated with different concentrations of sulfuric acid was investigated during enzymatic hydrolysis at a crude cellulase loading of 80 PCU/g AP-MWOP. The optimal incubation time for enzymatic hydrolysis was also evaluated at different incubation periods of 20, 30, 40, 50, and 60 min.

### 3.3. Enzymatic and Acid Hydrolysis of AP-MWOP

The AP-MWOP at a concentration of 50 g/L in AM1 medium was hydrolyzed with the crude cellulase enzyme in a 250 mL Erlenmeyer flask with a working volume of 50 mL. The VRE P3 crude cellulase is commercially available and was purchased from Siam Victory Chemicals Co., Ltd. (Bangkok, Thailand). According to the manufacturer’s instruction, the crude cellulase used in this study was derived from mixed cultures of *Trichoderma reesei* and *Bacillus subtilis*. This enzyme has an activity of >28,000 PCU/g enzyme, possessing combined activities of mainly cellobiohydrolase (exoglucanase) and endoglucanase, and trace xylanase. The crude cellulase was used to hydrolyze AP-MWOP at enzyme loadings of 60, 80, 100, 120, and 140 PCU/g AP-MWOP for 120 h. One PCU (protein centered unit) is defined as the amount of a combined enzymatic activity of cellobiohydrolase (exoglucanase) and endoglucanase that releases 1 nmol of 4-methylumbelliferone from 4-methylumbelliferyl-β-D-galactoside per second [48]. The condition of enzymatic hydrolysis was performed at 50 °C and pH 5.0 with a shaking speed of 200 rpm to facilitate the saccharification of AP-MWOP. The cellulase concentration resulting in the highest level of liberated fermentable sugars released from AP-MWOP was determined to be the most suitable enzyme loading for further experiments.

The complete saccharification of the AP-MWOP by strong acid digestion was adapted from the method of Dunning and Dallas [49]. The AP-MWOP at a weight of 1.0 g was mixed with 10 mL of warm 72% (*v*/*v*) H_2_SO_4_ (55 °C), and the slurry was agitated for 5 min. DI water was added to the slurry, reaching a volume of 200 mL, and the mixture was heated at 121 °C for 25 min. The sample was centrifuged, and the supernatant was prepared for HPLC analysis to determine the liberated sugar content.

### 3.4. Succinic Acid Production from AP-MWOP via Different Fermentation Modes

AP-MWOP pulping was used as a substrate for succinic acid production in a 5 L bioreactor (Infors, Basel, Switzerland) with a working volume of 2.5 L. The separate hydrolysis and fermentation (SHF) process was initially applied by hydrolyzing AP-MWOP using the VRE P3 cellulase enzyme at the optimized loading. Saccharification through SHF was carried out at 50 °C with agitation at 400 rpm. After 48 h of saccharification, the mixture was cooled to 37 °C at 200 rpm, and potassium bicarbonate at a final concentration of 100 mM was added to the fermenter. The seed inoculum of *E. coli* KJ122 was added to the slurry with an initial OD_600_ of 0.5. Fermentation began upon inoculation and was maintained at 37 °C. The pH of the fermentation broth was maintained at 7.0 by adding a mixed solution of 6 M KOH and 3 M K_2_CO_3_ at a 1:6 ratio. Different concentrations of AP-MWOP (50, 70, and 100 g/L) were used to determine the optimal substrate level for succinic acid production. Various agitation speeds (100, 200, and 300 rpm) were also optimized to test the effect of agitation speed on succinic acid production with the appropriate concentration of AP-MWOP.

AP-MWOP was also used as a substrate for succinic acid production during simultaneous saccharification and fermentation (SSF), following the same strategy as the SHF process mentioned above. However, the seed culture and the VRE P3 cellulase were added simultaneously. Fermentation was also performed under the same conditions as the SHF process. For fed-batch SSF, the optimal conditions from the batch experiments were applied to increase the succinic acid concentration, yield, and productivity in fed-batch mode. The pre-saccharification time was 0, 6, 12, 18, or 24 h before performing the fed-batch SSF process. AM1 medium containing 70 g/L AP-MWOP was initially utilized for succinic acid production. When the total glucose concentration in the fermentation broth decreased below 10 g/L, additional substrates were intermittently fed into the fermenter until the final glucose concentration reached 20–25 g/L. All the experiments were performed in triplicate.

### 3.5. Analytical Methods

The fermentation broths were collected every 6 h to quantify the cell mass, organic acids, and glucose. Cell growth was measured via a Bausch & Lomb Spectronic 70 spectrophotometer at OD_600_ nm and converted to biomass as the cell dry weight (1 OD_600_ = 0.333 g CDW/L biomass). High-performance liquid chromatography (HPLC) with an Aminex HPX-87H column (7.8 × 300 mm, Bio-Rad, Hercules, CA, USA) and refractive index (RI) detectors (RI-150, Thermo Spectra System, San Jose, CA, USA) were used to determine the organic acid and glucose contents throughout fermentation. The mobile phase in the HPLC system was sulfuric acid (4 mM) at a flow rate of 0.4 mL/min. The fermentation culture was centrifuged to separate the cells and AP-MWOP pulp from the supernatant. To prepare the sample, a tenfold dilution of the supernatant sample was performed with 20 mM H_2_SO_4_, and the diluted liquid was filtered through a 0.22 µm nylon filter prior to HPLC injection. The concentrations of sugars and organic acids were used to calculate the sugar consumption rate, succinate yield, and productivity. HPLC profiles of fermentation broths from the pre-saccharified fed-batch SSF process were provided as Figure S1. The number of bacterial cells that survived during fermentation was also measured by spreading the diluted broth on solid LB agar media, and the plates were then incubated at 37 °C for 24 h. The number of bacterial colonies that grew on the plates was then counted and reported as the number of colony-forming units per milliliter of culture (CFU/mL).

### 3.6. Statistical Analysis

One-way analysis of variance (ANOVA) was conducted via GraphPad/Prism 8.0.2 (GraphPad Software, Boston, MA, USA). Three independent replications were performed for each test, and average values are reported with standard deviations (SD). The differences among the mean values were established via Tukey’s test at the 95% significance level (*p* < 0.05).

## 4. Conclusions

A large amount of mixed waste office paper (MWOP) is generated globally due to economic growth in every sector, but recycling it is costly. This study represents an effective alternative means to valorize cellulose and hemicellulose contents in MWOP for high-value bio-succinic acid production by *E. coli* KJ122. The fed-batch SSF process significantly improved succinic acid production to 51.3 g/L, with yields of up to 0.75 g/g sugars utilized or 0.04 g/g AP-MWOP provided. This work allows the reuse of MWOP to produce succinic acid as a viable and efficient method to promote a green and zero-waste society.

## Figures and Tables

**Table 1:** Comparison of succinic acid production from pretreated AP-MWOP under different fermentation conditions.

Fermentation Condition	Total Time (h)	Succinic Acid (g/L)	Conversion Yield * (g/g)	Gross Yield (g/g)	Productivity (g/L/h)	Acetic Acid (g/L)
SHF						
50 g/L	72	21.79 ± 0.65 ^d^	0.90 ± 0.02 ^a^	0.43 ± 0.01 ^a^	0.90 ± 0.02 ^ab^	4.10 ± 0.10 ^bc^
70 g/L	72	28.19 ± 0.98 ^c^	0.87 ± 0.03 ^a^	0.40 ± 0.02 ^ab^	1.17 ± 0.04 ^a^	5.36 ± 0.31 ^b^
100 g/L	102	36.62 ± 0.02 ^b^	0.73 ± 0.01 ^b^	0.36 ± 0.01 ^b^	0.67 ± 0.01 ^b^	7.15 ± 1.12 ^b^
Pre-saccharified SSF						
50 g/L + 0 h pre-saccharification	48	14.51 ± 1.15 ^e^	0.58 ± 0.05 ^c^	0.29 ± 0.02 ^bc^	0.29 ± 0.02 ^c^	4.70 ± 0.15 ^bc^
50 g/L + 6 h pre-saccharification	42	15.36 ± 1.03 ^e^	0.61 ± 0.04 ^c^	0.30 ± 0.02 ^bc^	0.42 ± 0.02 ^bc^	5.91 ± 0.91 ^b^
50 g/L + 12 h pre-saccharification	48	17.15 ± 0.03 ^e^	0.68 ± 0.01 ^bc^	0.34 ± 0.01 ^b^	0.47 ± 0.02 ^bc^	3.67 ± 0.14 ^bc^
50 g/L + 18 h pre-saccharification	48	22.44 ± 1.93 ^d^	0.89 ± 0.07 ^a^	0.44 ± 0.03 ^a^	0.74 ± 0.04 ^bc^	5.64 ± 0.07 ^b^
50 g/L + 24 h pre-saccharification	48	21.39 ± 0.01 ^d^	0.85 ± 0.01 ^a^	0.42 ± 0.01 ^a^	0.71 ± 0.04 ^ab^	5.61 ± 1.23 ^b^
70 g/L + 18 h pre-saccharification	48	24.58 ± 2.32 ^d^	0.76 ± 0.07 ^ab^	0.35 ± 0.03 ^b^	0.82 ± 0.07 ^ab^	7.25 ± 1.19 ^b^
Fed-batch SSF	66	51.38 ± 4.05 ^a^	0.75 ± 0.05 ^b^	0.04 ± 0.03 ^d^	1.07 ± 0.08 ^a^	11.47 ± 1.98 ^a^

* Conversion yield is defined as gram succinic acid produced divided by gram total sugars consumed, while gross yield is defined as gram succinic acid produced divided by gram AP-MWOP. Different superscript lower-case letters indicate significant differences between mean values (*p* ≤ 0.05) in the same column.

## Data Availability

All data generated or analyzed during this study are included in this published article.

## Supplementary Materials

The following supporting information can be downloaded at https://www.mdpi.com/article/10.3390/ijms26030982/s1.
