# Supplementary material for: Exploiting Mixed Waste Office Paper Containing Lignocellulosic Fibers for Alternatively Producing High-Value Succinic Acid by Metabolically Engineered Escherichia coli KJ122
Source: Int J Mol Sci. 2025 Jan 24;26(3):982. doi: 10.3390/ijms26030982 (PMC11817568; doi:10.3390/ijms26030982)
Supplement: Supplementary file 1 [file ijms-26-00982-s001.zip › ijms-3422379-supplementary.pdf]

*Supplementary Materials*

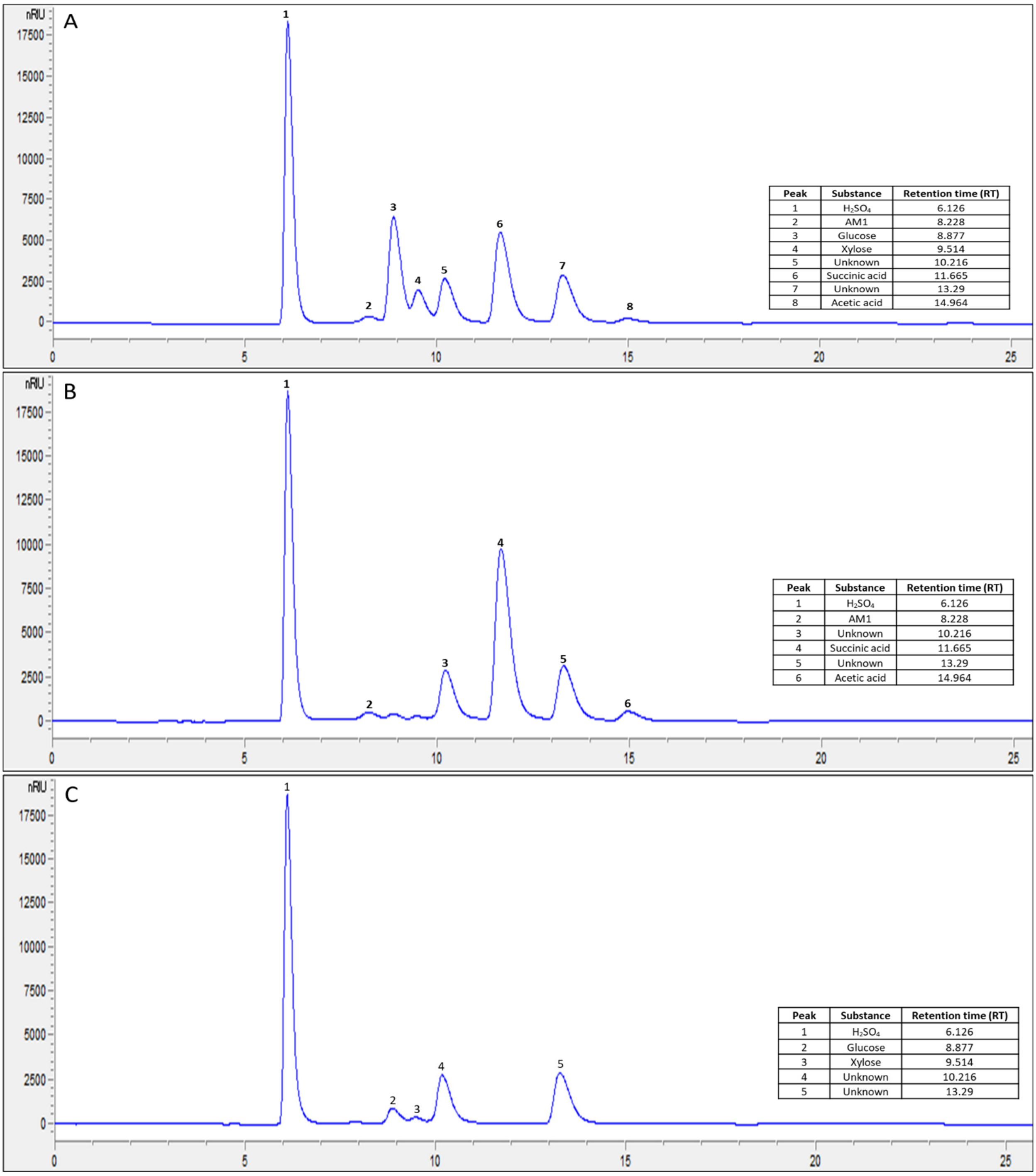


**Figure S1.** HPLC profiles of fermentation broth of succinic acid production from AP-MWOP by *E. coli* KJ122 via a pre-saccharified fed-batch SSF process. (**A**) 30 h, (**B**) 54 h, and (**C**) VRE P3 crude cellulase.
